# “A Bridge-over-the Bar”: A Novel Strategy to Prevent Paravalvular Regurgitation during Mitral Valve Replacement for Severe Mitral Annular Calcifications

**DOI:** 10.5761/atcs.nm.24-00081

**Published:** 2025-01-11

**Authors:** Khaled F. Salhab, Sameh M. Said

**Affiliations:** 1 Department of Cardiothoracic Surgery, New York University Langone, Long Island, NY, USA; 2 Division of Pediatric and Adult Congenital Cardiac Surgery, Maria Fareri Children’s Hospital, Department of Surgery, Westchester Medical Center, New York Medical College, Valhalla, NY, USA

**Keywords:** mitral annular calcification, periprosthetic mitral regurgitation, paravalvular leak, mitral valve replacement

## Abstract

Mitral annular calcifications have been known to increase complexity during mitral valve replacement (MVR). Standard procedure requires decalcification followed by reconstruction of the mitral annulus prior to placing the prosthesis. While this is the ideal technique, it is not feasible in every patient due to the associated risks. The mere attempt at valve replacement without proper annular decalcification has been associated with a high incidence of periprosthetic leak which complicates the postoperative course and has been associated with increased morbidity and mortality. With the advances in transcatheter therapy, postoperative periprosthetic regurgitation can be managed with devices and primary transcatheter valve implantation could be alternative to standard valve replacement; however, these alternate strategies are not without its own limitations and drawbacks. In the current report, we present a novel strategy to be used in a select group of patients with severe but non-circumferential annular calcifications to prevent/minimize periprosthetic regurgitation during MVR. This involves placing a patch over the posteriorly located calcium bar, thus minimizing tension on the posterior suture line and contain any periprosthetic regurgitation if to develop. This modification has been performed in a total of nine cases with acceptable early results.

## Introduction

Mitral annular calcifications (MAC) are known risk factors for worse outcomes during standard mitral valve replacement (MVR).^1)^ Several transcatheter, surgical and/or hybrid techniques have been proposed to handle MAC.^2)^ The ideal and standard technique involves complete decalcification of MAC, followed by reconstruction of the left atrioventricular junction with a patch and placement of the mitral prosthesis.^3)^ This technique is rather complex and requires long ischemic and cardiopulmonary bypass times which have been associated with increased morbidity if not mortality. Not every patient is a good candidate for such long and complex reconstruction, therefore alternate strategies must exist.

With the advances in transcatheter therapy, transcatheter valve-in-MAC has been a good alternative to minimize risks associated with the standard surgical technique. This technique, however, requires proper patient selection to ensure its success.^4)^ Ideal candidates have been the ones with complete/circumferential MAC and with low risk of development of left ventricular outflow tract (LVOT) obstruction.

For those who can be considered intermediate risks, a hybrid strategy may be selected, in which a modified transcatheter prosthesis can be implanted via a direct trans-atrial approach.^5)^ In a select group of patients who have severe posteriorly located MAC, the above-mentioned strategies have their limitations and drawbacks.

We propose a new and modified approach to standard MVR in these patients to prevent/minimize the occurrence of paravalvular leak (PVL) in the presence of posterior MAC.

## Technique

Cardiopulmonary bypass is initiated in the standard fashion using aortic and bicaval cannulation. Cardioplegic arrest is then achieved with antegrade cardioplegia. Standard vertical left atriotomy has been our main approach to the mitral valve in these cases and usually the left atrium is enlarged in these patients which provides adequate exposure to the mitral valve.

**Fig. 1:**
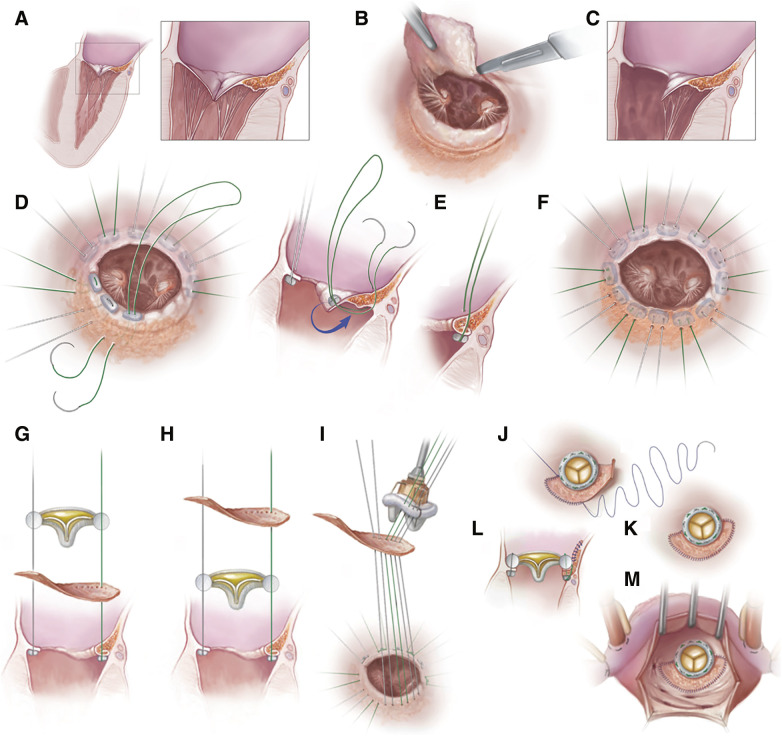
Illustrations showing the details of our technique: (**A**) It is more applicable to posterior annular calcifications with or without extension to the posterior leaflet (notice the close relationship of the posterior calcium bar to the circumflex vessels), (**B** and **C**) the anterior leaflet is resected and the posterior leaflet is retained with its related subvalvular apparatus if possible to preserve the left ventricular function, (**D**) standard multiple interrupted pledgeted sutures are placed in a horizontal mattress fashion around the mitral annulus (pledgets are placed on the ventricular side), (**E**) the sutures placed posteriorly in a way to fold the posterior mitral leaflet and then passed through the posterior calcium bar with caution to avoid injuring the circumflex coronary vessels, (**F**) final appearance of the sutures after completion. (**G**) the sutures are then passed through the prosthesis sewing ring in the standard fashion, however in the area of the calcium bar/posterior annulus, the sutures pass through the prosthesis ring followed by the pericardial patch (**H**) or can pass through the pericardial patch prior to the prosthesis sewing ring (**I**). (**J**) Once the prosthesis is secured in position and sutures are tied, the patch is sewn to the left atrial wall above and away from the calcium bar. (**K**) This is done using running prolene suture. (**L**) a side view showing the principle of the technique in which the calcium bar is now sandwiched between the pledgeted sutures and the folded posterior leaflet on one side and the pericardial patch on the other. (**M**) Final appearance from the surgeon’s view through the opened left atrium where the prosthesis is well-seated and the patch is secured to the left atrial wall bridging over the posteriorly located calcium bar.

Initial assessment confirms the preoperative cross-sectional and echocardiographic images and determines the extent and degree of MAC (**Fig. 1A**). The anterior mitral leaflet is usually resected (**Fig. 1B**), and steps are taken to preserve the posterior leaflet and subvalvular apparatus if possible (**Fig. 1C**). Standard pledgeted braided sutures are placed at the native mitral annulus with the pledgets on the ventricular side (**Fig. 1D**). At the posteriorly-located MAC bar, the sutures are placed with a larger needle that passes through the posterior leaflet or through/around the MAC bar taking in consideration the location of the circumflex coronary artery (**Fig. 1E**). The calcium bar in these patients most of the time will allow the passage of the needle due to its caseous content, so there are areas that are relatively soft and will permit the passage of the needle. This also explains why this suture line may dehisce resulting in PVL. From a practical stand point, the posterior suture line is created by passing the needle through any area along the posterior annulus that permits the needle pass. The sutures then pass through the sewing ring of the prosthesis which is modified by sewing a bovine pericardial patch to its posterior sewing ring (**Fig. 1F**).

**Fig. 2:**
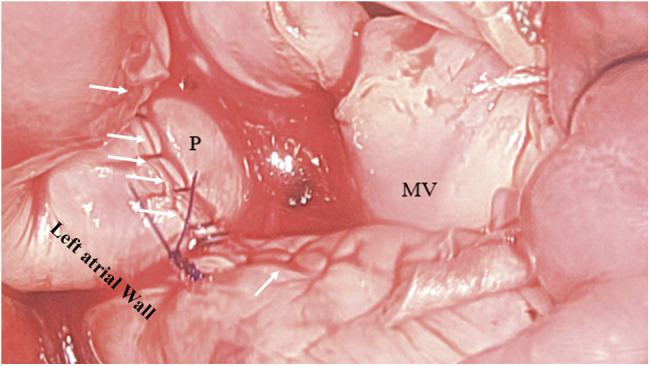
Intraoperative photo (close-up view) through the opened left atrium showing the final appearance of the new mitral prosthesis in position (MV) and the completed patch (P) that has been secured to the left atrial wall with running suture (multiple white arrows). MV: mitral valve prosthesis; P: patch

The patch is fashioned in a way that depends on the extent of the posterior MAC bar. Its length is the extent of the MAC posteriorly and its width is the posterior extent of the calcium bar (**Figs. 1G–1I** and **2**). The prosthesis is then secured in position, taking caution not to tear the posterior suture line (**Figs. 1J** and **1K**). The patch is then secured to the left atrium with running polypropylene sutures and can be further reinforced with additional pledgeted sutures if needed (**Figs. 1L** and **1M**). The rest of the procedure is then completed in the standard fashion.

## Authors’ Experience and Outcomes

**Fig. 3:**
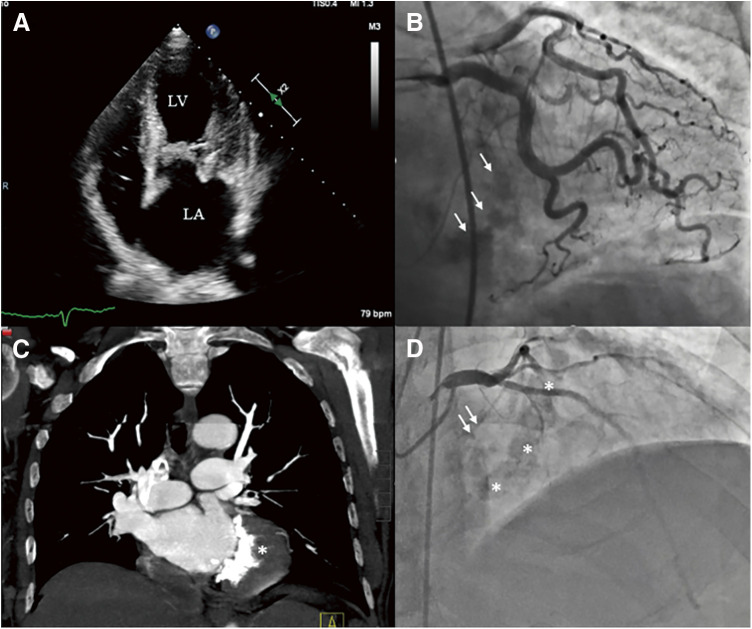
Preoperative imaging studies of different patients in which the current technique was successfully performed showing (**A**) echocardiogram with severely thickened native mitral valve and posterior annular calcifications, (**B**) preoperative coronary angiogram showing the posterior mitral annular calcification (multiple white arrows), (**C**) preoperative computed tomography scan showing severe mitral annular calcification (asterisk), and (**D**) preoperative coronary angiogram showing moderate degree of posterior mitral annular calcification (multiple asterisks) with minimal anterior calcifications (two white arrows). LA: left atrium; LV: left ventricle

The combined authors’ experience includes 9 patients with varying degrees of posterior MAC (**Figs. 3A–3D**) and minimal to no anterior annular calcifications. They all shared the lack of candidacy to transcatheter valve-in-MAC and were considered high risk candidates for mitral annular decalcification, annular reconstruction and standard MVR.

The procedure was successful in all patients with no mortality. One patient developed PVL in the follow-up period and was managed with transcatheter device closure of the leak site. A second patient developed low cardiac output postoperatively and was taken for emergency coronary angiography to rule out any compromise to the circumflex coronary artery which was found patent and he recovered prior to hospital discharge.

## Discussion

Periprosthetic regurgitation has been the Achilles’ heel of MVR in the presence of MAC.^6)^ Several classifications have been proposed for MAC that are based on preoperative cross-sectional imaging and intraoperative assessment. In our opinion, classifying MAC is important as it determines the best approach for MVR in these patients.

Several strategies are available which allow MVR in the presence of MAC. These include complete debridement with removal of the calcium bar followed by reconstruction of the left atrioventricular grove and standard prosthesis placement, direct suture placement through the MAC, translocating the prosthesis to the left atrium (supra-annular placement of the prosthesis) which has been associated with risk of left atrial dissection,^7)^ mitral valve bypass,^8)^ direct trans-atrial implantation^5)^ or totally transcatheter techniques.^4)^ As discussed previously, while the ideal technique is proper and complete annular decalcification followed by annular reconstruction and valve replacement, it cannot be performed for every patient and proper selection is a key to ensure successful MVR with minimal morbidity and mortality. Transcatheter valve-in-MAC requires circumferential MAC to ensure proper placement of the prosthesis and has been associated with the risk of LVOT obstruction.

A select group of patients remains in need for MVR, but they are not good candidates for either of the above-mentioned techniques. This group includes those with posteriorly located MAC. The current technique will not be suitable for those with circumferential MAC or MAC that extends to the posterior left ventricular wall and papillary muscles. These cases will not allow placing a standard prosthesis without debridement and decalcification of the mitral annulus. But it can be used as a complementary technique for some of these cases as well to add an additional layer of protection against posterior PVL.

It is important to understand that placing the posterior suture line for the prosthesis through the posterior mitral leaflet or through/around the posterior calcium bar is a technqiue that is frequently done by many surgeons to avoid dealing with the calcium bar, however, its main drawbacks is the breakdown in the calcium bar or posterior leaflet, resulting in PVL. Our patch modification protects that posterior suture line and serves two important functions: decrease tension on the posterior suture line and contain the leak if developed.

There are several advantages to our proposed technique. This includes its simplicity and reproducibility. It avoids the challenges and complexity associated with complete annular decalcification as well as the risks involved, mainly atrioventricular grove disruption. It also addresses the PVL that may occur with standard placement of the valve sutures along the posterior calcium bar by creating a patch over that bridge of calcium which helps limit the PVL to the area under the patch. This acts as a safety mechanism to secure the prosthesis with minimal tension on the posterior annulus as well as the left atrium. An important tip in these cases is that we intentionally chose a prosthesis with a smaller sewing cuff (low profile) which adds the advantage of allowing a bigger prosthesis size placement in a relatively tight space and minimizes its interaction with the calcium bar. One should also notice that the atrial side of the calcium bar is not smooth but in other terms, the patch can be considered as a posterior extension to the prosthesis sewing ring.

The left atrium in these patients is usually heavily muscularized due to the long-standing severe mitral stenosis so securing the patch posteriorly to the left atrium should not be associated with increased risk of dehiscence compared to sewing the left atrial wall to the prosthesis sewing ring directly which could lead to left atrial wall dissection as has been reported previously.^7)^

The current report represents an early experience with this technique, and longer follow-up is needed to determine its durability.

## Conclusions

We believe the current modification is simple, reproducible and is associated with minimal risks. It should be considered in this selected group of patients whom are not suitable candidates for transcatheter therapy and/or standard mitral annular decalcification and reconstruction. Longer-term follow-up will be needed to determine its durability.

## Declarations

### Ethics approval and consent to participate

A specific institutional review board (IRB) approval was not required for the current technique due to the nature of the report, however permission was obtained from the patients we used their images/studies to support the current report.

### Consent for publication

Permission was obtained from patients to use their imaging studies to support the current report.

### Funding

No funding.

### Data availability

Data are available up on request to the corresponding author.

### Authors’ contributions

Khaled F. Salhab: review and editing

Sameh M. Said: the concept of the technique and writing the initial draft

Both authors used the described technique in their clinical practice. All authors have read and approved the manuscript, and they are responsible for the manuscript.

### Disclosure statement

The authors have no conflict of interest to disclose with the current report. However, the author (S.M.S) is a consultant for Artivion.
